# Assessment of quality of life using the EQ-5D-3L instrument for hospitalized patients with femoral fracture in Brazil

**DOI:** 10.1186/s12955-018-1017-5

**Published:** 2018-09-24

**Authors:** Ivanise Arouche Gomes de Souza, Claudia Cristina de Aguiar Pereira, Andrea Liborio Monteiro

**Affiliations:** 1 Instituto Nacional de Traumatologia e Ortopedia – INTO, Avenida Brasil n° 500, 9° andar - sala GRISC, São Cristóvão, Rio de Janeiro, RJ CEP: 20940-070 Brazil; 2 FIOCRUZ, Escola Nacional de Saúde Pública, Rua Leopoldo Bulhões 1480 - sala 708, Manguinhos, Rio de Janeiro, RJ CEP 21041-210 Brazil; 3 College of Pharmacy, University of Illinois at Chicago, 833 S. Wood St, Chicago, IL 60612 USA; 4 Instituto Nacional de Cardiologia, Nucleo de Avaliacao de Tecnologias em Saude, Rua das Laranjeiras 374, Rio de Janeiro, RJ Brazil

**Keywords:** EUROQoL, EQ-5D-3L, VAS, Femoral fracture, Quality of life

## Abstract

**Background:**

Quality of life has become a key outcome in assessing the effectiveness of treatments and interventions in health.

**Methods:**

Accordingly, this research study aimed to measure quality of life using the EQ-5D-3L instrument for patients from the Jamil Haddad National Institute of Traumatology and Orthopedics (Instituto Nacional de Traumatologia e Ortopedia Jamil Haddad – INTO) with femoral fractures, hospitalized between 11/2015 and 10/2016.

**Results:**

A total of 165 orthopedic trauma patients with femoral fractures, aged 18 years or older, who were hospitalized and operated upon in the INTO were assessed. The assessment instruments were applied at admission and in the first and second follow-up visits to the outpatient clinic. Most study subjects were women and older than 60 years. Proximal femoral fracture was the most commonly found fracture. The Visual Analog Scale (VAS) assessments over the study period showed an increasing gain in self-assessed quality of life. Similarly, the EQ-5D-3L showed significant improvements in quality of life assessed in the five dimensions of the instrument: mobility, self-care, usual activities, pain/discomfort and anxiety/depression.

**Conclusion:**

This type of assessment may help in decision-making and cost-utility assessments related to orthopedic trauma.

## Background

In Brazil, approximately 90,000 femoral fractures occur per year, with an annual cost (considering only the surgical procedures) of approximately 70 million Brazilian reals for the healthcare system [1]. In addition to the economic and social damage caused by femoral fractures, this type of fracture has a negative impact on the quality of life of patients and causes a high mortality rate due to post-fracture or postoperative complications.

Another worrying factor regarding femoral fractures among the elderly is the large number of associated chronic diseases. A total of 70% of elderly patients have at least two other diseases at the time of the fracture and are, thus, much more vulnerable to complications in both the immediate and late postoperative periods [2].

Studies in developed countries suggest that some preoperative factors are more associated with increased mortality in patients with femoral fracture at admission, such as age and clinical comorbidities [3].

Thus, femoral fractures remain an important public health problem and one of the most common and devastating traumatic lesions among the geriatric population [4]. They mainly occur in the proximal region and may be associated with high- and low-energy trauma, usually resulting from falls, which are more common among elderly people. These fractures affect more women than men and, even when they are well consolidated, are associated with high morbidity and mortality rates [5, 6].

The rate of mortality due to femoral facture among elderly people ranges from 20 to 30% on average and may reach 50% before the end of the first year, according to a research study conducted by Garci, Leme & Leme [7]. The following complications are among the causes of death: pneumonia, pulmonary embolism and thrombosis [8].

Thus, considering the importance of femoral fractures for society and the impact on the lives of individuals with these fractures, this research study focused on assessing the quality of life of patients with these types of fractures.

This research study aimed to compare the measurements of quality of life taken at different times of the femoral fracture treatment (at hospitalization for orthopedic surgery; at the first visit after the surgery for stich removal and at the second follow-up visit); to identify sociodemographic, clinical and surgical factors related to quality of life during the study period; and, lastly, to calculate the utility measures associated with the different fracture types of the sample.

The concept of “quality of life” was initially studied by social scientists, philosophers and politicians. However, considering multiple factors, such as the progressive dehumanization of healthcare systems resulting from the accelerated technological development of medicine, the focus of the *“quality of life”* assessment was broadened towards quantifying the impacts of diseases and treatments on the routine and functional capacity of patients, going beyond symptom control, decreased mortality or increased life expectancy [9].

## Methods

Orthopedic trauma patients with femoral fractures, aged 18 years or older, who were hospitalized and treated in the Jamil Haddad National Institute of Traumatology and Orthopedics (Instituto Nacional de Traumatologia e Ortopedia Jamil Haddad – INTO) were recruited for the study sample. Patients with femoral fractures admitted to the INTO were included in the sample. Patients with other fractures in addition to the femoral fracture were excluded from the sample. We also excluded from our sample all patients with cognitive problems according to their medical records.

The fractures were classified as to the type of fracture using the Arbeitsgemeinschaft für Osteosynthesefragen [Association for the Study of Internal Fixation]/ Orthopaedic Trauma Association (AO/OTA) Fracture and Dislocation Classification [10] and were grouped into proximal, diaphyseal and distal fractures for analysis purposes. It should be noted that in the total of 165 participants composing the sample, not all patients were classified according to the AO/OTA system. Thus, 17 patients were classified according to different systems, such as: Tronzo, Gartland and Schatzker, as they were more suitable to those cases. However, they remained in the study and were also assessed at three time points to follow the progression or regression in quality of life.

The data were collected through face-to-face and telephone interviews (conducted when a patient missed an appointment) by the researcher and through searches in the medical records. Patients were invited to participate in the study upon admission to the Institute. The interviews were performed after explaining the purpose of the study to the patients, who signed the Informed Consent Form (Termo de Consentimento Livre e Esclarecido; supplemental material). Ethics Committee Approval Number: CAAE 50507715.3.0000.5273.

A quality of life instrument, based on economic theory and able to calculate the quality-adjusted life year (QALY), termed the EuroQol five-dimensions-three-level questionnaire (EQ-5D-3L) [11], was used for data collection. This questionnaire consists of a descriptive system that assesses quality of life based on health states through questions regarding five basic health and functional dimensions: mobility, self-care, usual activities, pain discomfort and anxiety/depression. Each domain is divided into three levels of perceived problems indicated in the answers: the individuals are asked if they have no problems (1), some problems (2) or extreme problems (3). The answers provided for the five dimensions are then converted into a summary score, which indicates the overall utility.

For each individual, their answer to the descriptive system generates a five-digit code corresponding to the level of perceived problems indicated in the answers to the five questions regarding the five dimensions of the instrument. Because each question may have three mutually exclusive answers, the possible combinations of answers to the EQ-5D-3L result in 243 health states described by this questionnaire [11]. The use of the EQ-5D has been recommended to measure outcome for patients recovering from hip fracture [12, 13]. The weight applied to the states is based on the national validation study of the EQ-5D conducted by the Health Technology Assessment Group (Núcleo de Avaliação de Tecnologias em Saúde – NATS)/ National Institute of Cardiology (Instituto Nacional de Cardiologia – INC).

The data collection instrument also consisted of a visual scale, termed the Visual Analog Scale (VAS), which enables the respondent to self-assess their health state on a scale graded from 0 to 100, where 0 corresponds to the “worst imaginable health state” and 100 to the “best imaginable health state”.

The results were pooled towards making the graphical representations of the results from the application of the VAS instrument [14], more didactic, as described below. Based on this classification, the participants self-reported their health state as Very Good (81–100), Good (51–80), Normal (31–50) and Bad/Very Bad (0–30).

The instrument was applied upon patient admission, which was considered Time Zero (t_0_), when we first contacted the patient, and eligibility was assessed to form the sample of this study. After inclusion in the study, we reapplied the same instrument in the first follow-up visit of this patient to the outpatient clinic, at the time point then termed Time 1 (t_1_): at this time, the researcher actively searched for the appointment in the medical consultation system. In the second follow-up visit, Time 2 (t_2_), the patient returned to the outpatient clinic, without an appointment at a predetermined time, when we reapplied the instrument and completed the follow-up. It should be noted that the researcher read the questionnaire out loud to patients with reading difficulties or who were illiterate, subsequently recording their answers on the sheet.

Data collection was divided into three parts: the first consisted of patient identification information, such as name, age, marital status, education, profession, family income, religion and address. In the second part, data on hospitalization and clinical conditions of the patient, date of hospitalization, date of fracture, date of surgery, date of hospital discharge, time of walking and the presence of comorbidities, such as diabetes, hypertension, urinary infection, chronic kidney disease, cancer, heart disease, rheumatoid arthritis, surgical and postoperative complications, weight and height were collected. The third part was the EQ-5D-3L instrument.

The participant was asked to choose the option that best described his or her health state in each of the five dimensions. This choice resulted in a single digit indicating the level selected for each dimension assessed. The digits resulting from the assessment of the five dimensions were combined into a 5-digit number describing the health state of the patient.

The data were analyzed using descriptive statistics, hypothesis tests and linear regression models and data from the three study periods: admission (t_1_), stich removal (t_2_) and second follow-up (t_3_). The sample was characterized using descriptive statistics. The utility values were estimated based on the validation study of the EQ- 5D-3L [15]. Given the non-parametric distribution of the outcome variable, a nonparametric test was used to compare the distribution of values between different assessment times (Friedman Test). For this purpose, head-to-head comparisons (t_1_ vs. t_2_, t_2_ vs. t_3_) were performed using the Wilcoxon signed-rank test with Bonferroni correction.

A linear regression model was constructed to identify the determinants of change in quality of life (gains and losses) between the t_1_ and the t_3_ assessments. The outcome variable (change in quality of life) of this regression was constructed by calculating the difference between the quality of life reported in the t_1_ and t_3_ assessments. The variables were selected for the model using the software package *vselect*. The statistical tests were performed using the statistical software STATA 14 (StataCorp LP, Stata Statistical Software; TX, USA) [16].

## Results

### Sample profile according to sociodemographic and health indicators

Over 11 months of data collection, 165 patients were included in the study, generating the sample profile of this research study outlined below in Tables 1 and 2.

**Table 1:** Sociodemographic and health characteristics of the orthopedic trauma sample of patients with femoral fractures – INTO, 2015–2016

Age group	Sample percentage		
18–24	4.3%		
25–60	35.0%		
61–79	37.0%		
≥ 80	23.7%		
Sex			
Male	46.7%		
Female	53.3%		
Education			
Illiterate	16.4%		
Primary education	55.1%		
Secondary education	22.4%		
Higher education	6.1%		
Family Income			
Less than 1 minimum wage	15%		
1 minimum wage	34%		
2 minimum wages	42%		
3 to 5 minimum wages	4%		
Higher than 5 minimum wages	3%		
Undisclosed	2%		
Self-assessed health (VAS)	VAS1	VAS 2	VAS 3
Bad/very bad (0–30)	8%	7%	11%
Normal (31–50)	41%	23%	12%
Good (51–80)	44%	61%	59%
Very good (81–100)	7%	9%	18%
Comorbidities	Number of People	Sample Percentage	
Urinary infection	26	15.8%	
Diabetes mellitus	36	21.8%	
Arterial hypertension	95	57.6%	
Cancer	11	6.7%	
Heart disease	31	18.8%	
Rheumatoid arthritis	7	4.2%	
Chronic kidney failure	18	10.9%

**Table 2:** Type of fracture occurred in orthopedic trauma patients with femoral fractures according to sex – INTO, 2015–2016

Type of fracture	Men		Women	
	No. People	Percentage	No. People	Percentage
Proximal	50	71.4%	60	76.9%
Diaphyseal	18	25.7%	9	116%
Distal	2	2.9%	9	11.5%

The results show that the age group most affected by femoral fractures in the study sample consisted of people older than 60 years; among those elderly people, this type of orthopedic fracture was more commonly found among women.

Furthermore, the most frequent fracture in the study sample was proximal femoral fracture, as shown in the table below. It should be noted that proximal femoral fractures are strongly related to ground-level falls, which are a common cause of hospitalization of elderly people in Brazil.

It should be noted that 10% of the total sample, according to the fracture classification, lacked surgical profiles. However, these patients remained as study participants, even after the decision to maintain the conservative fracture treatment.

### Assessment of self-assessed health using the Visual Analog Scale (VAS)

**Fig. 1:**
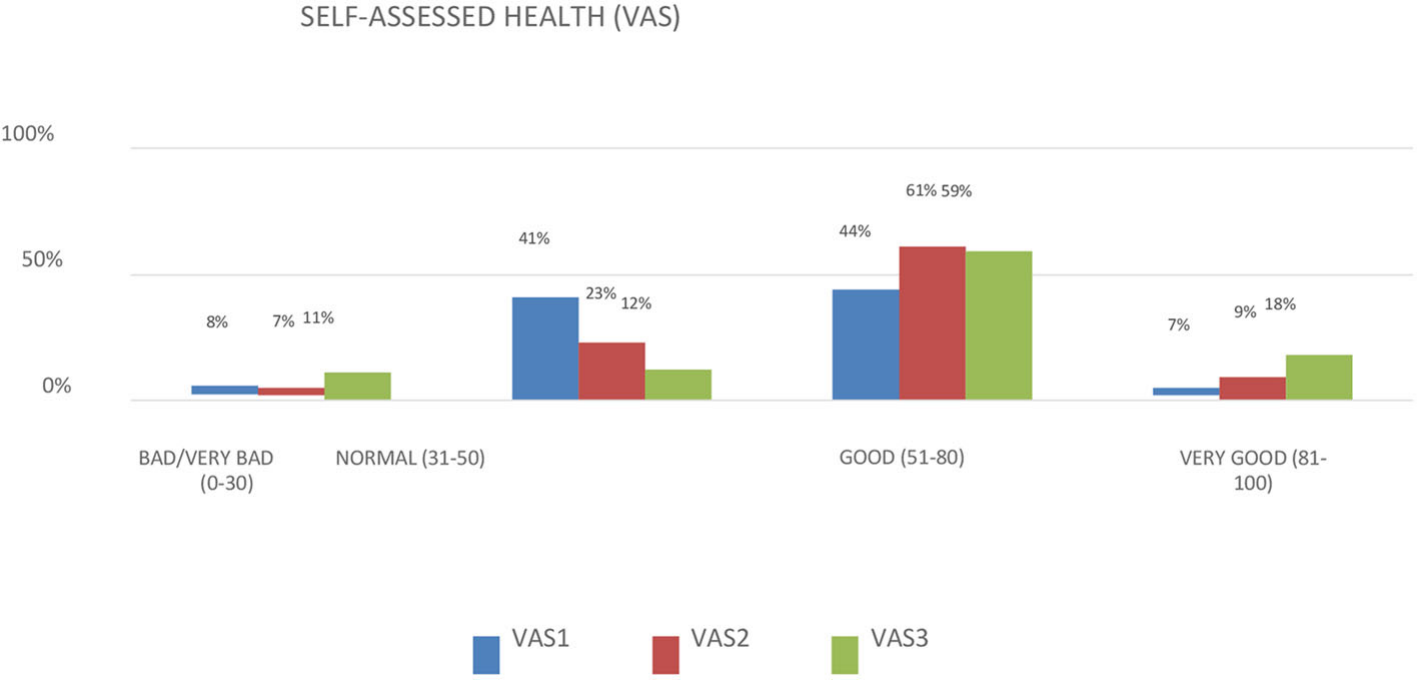
Self-assessed health. VAS1 – admission; VAS2 – first follow-up visit for stich removal; VAS3 – second follow-up visit in thperiod between 45 and 60 days after surgery

Health was self-assessed using the VAS at three time points: Admission - VAS1 and first - VAS2 and second - VAS3 follow-up visits. This assessment showed that, in general, the sample population tended towards a gain in quality of life after surgery. However, Fig. 1 shows an increase in the percentage of patients who reported bad self-assessed health states at VAS3 because of the number of deaths (08) that occurred during the study period.

### Assessment of self-reported quality of life using the EQ-5D-3L

**Fig. 2:**
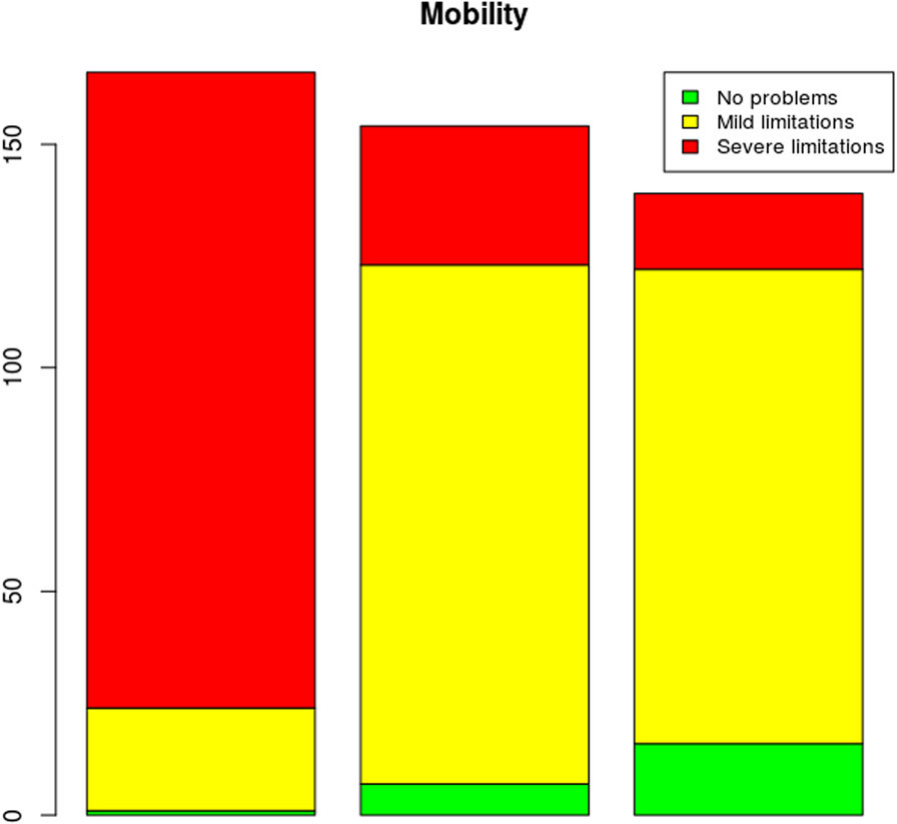
Problems reported in the mobility dimension at the three assessment times. The first time point was at admission, the second time point was at the first follow-up visit for stich removal, and the final time was at the second follow-up visit (45 to 60 days after the surgical procedure)

In this research study, the EQ-5D-3L instrument was used for assessing the health states of the sample population. For this purpose, the following dimensions were assessed: mobility; self-care; usual activities; pain/discomfort and anxiety/depression. These dimensions were also assessed using the VAS, at three time points, as shown in the following graphs:

As shown in Fig. 2, regarding the assessment of mobility conducted at three time points using the EQ-5D-3L instrument, in general, an improvement in patient mobility was observed when comparing times 1 and 3.

**Fig. 3:**
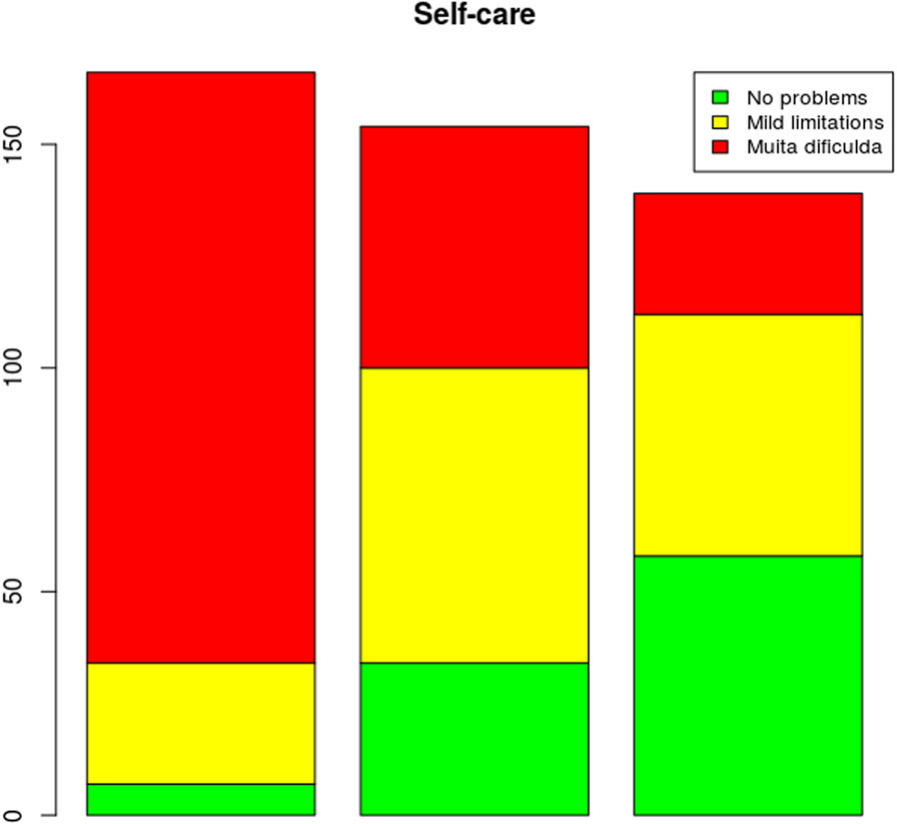
Problems reported in the self-care dimension at the three assessment times

Regarding the assessment of self-care (Fig. 3), the use of the EQ-5D-3L instrument to assess the patients at the three times showed a considerable decrease in the number of patients reporting extreme problems regarding self-care.

**Fig. 4:**
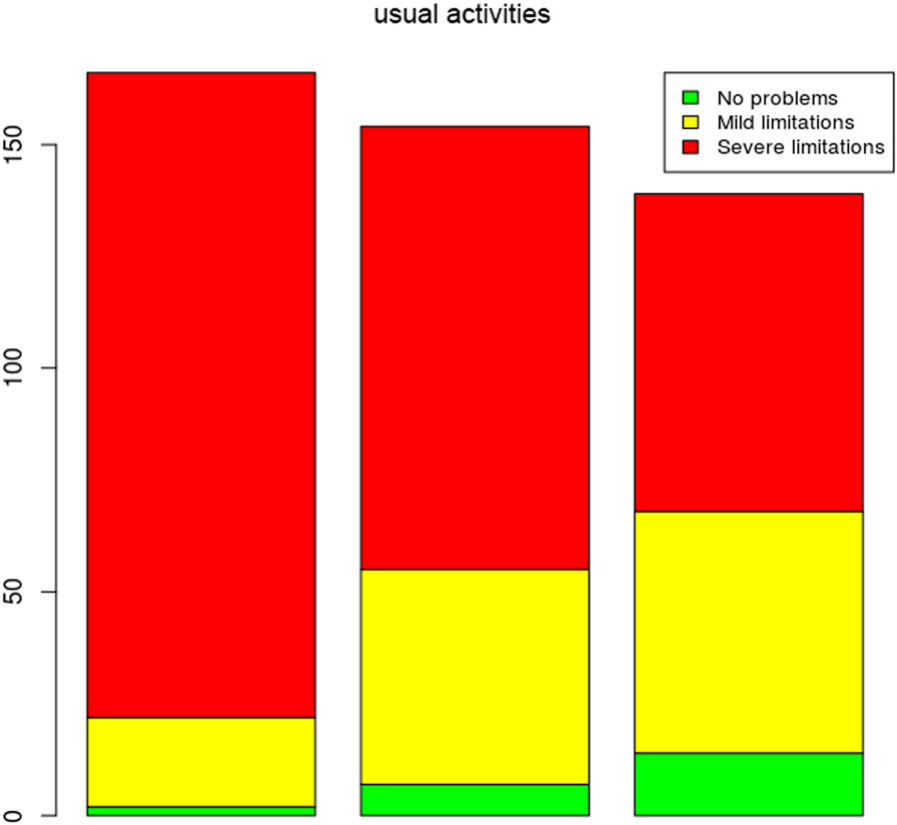
Problems reported in the usual activities dimension at the three assessment times

Regarding usual activities (Fig. 4), the analysis of the problems reported in this dimension between times 1 and 3 showed gains in usual activities throughout the treatment time.

**Fig. 5:**
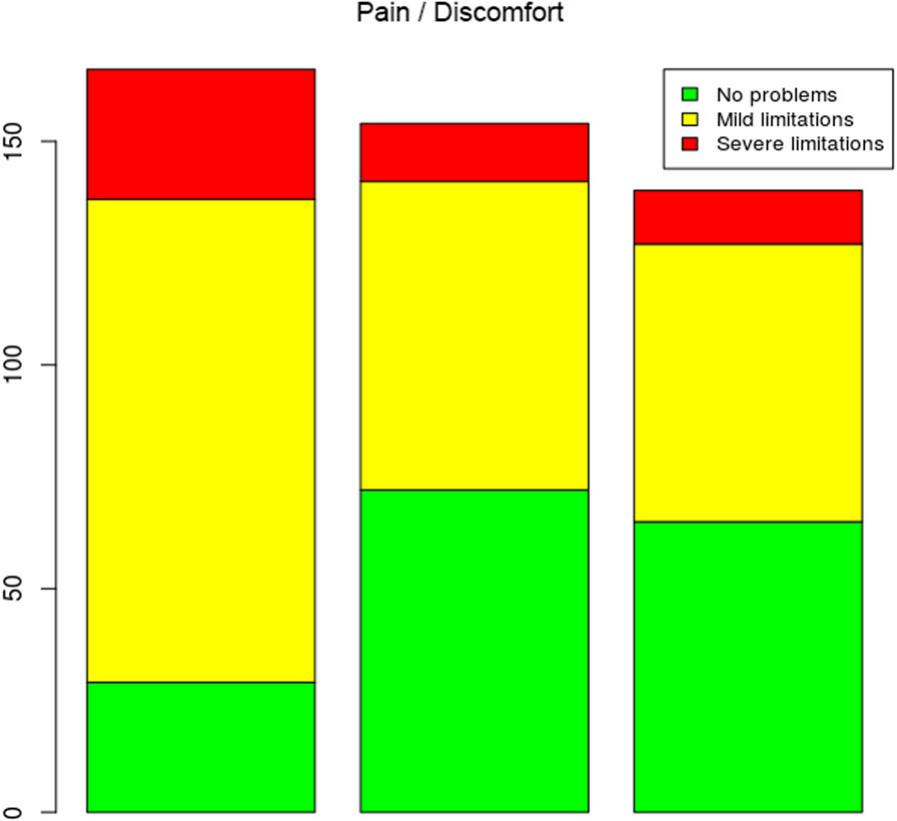
Problems reported in the pain/discomfort dimension at the three assessment times

Regarding the assessment of the pain/discomfort dimension (Fig. 5), the level of pain reported by the patients between times 1 (admission) and 2 (stich removal) improved. However, between times 2 and 3, this improvement was not maintained.

**Fig. 6:**
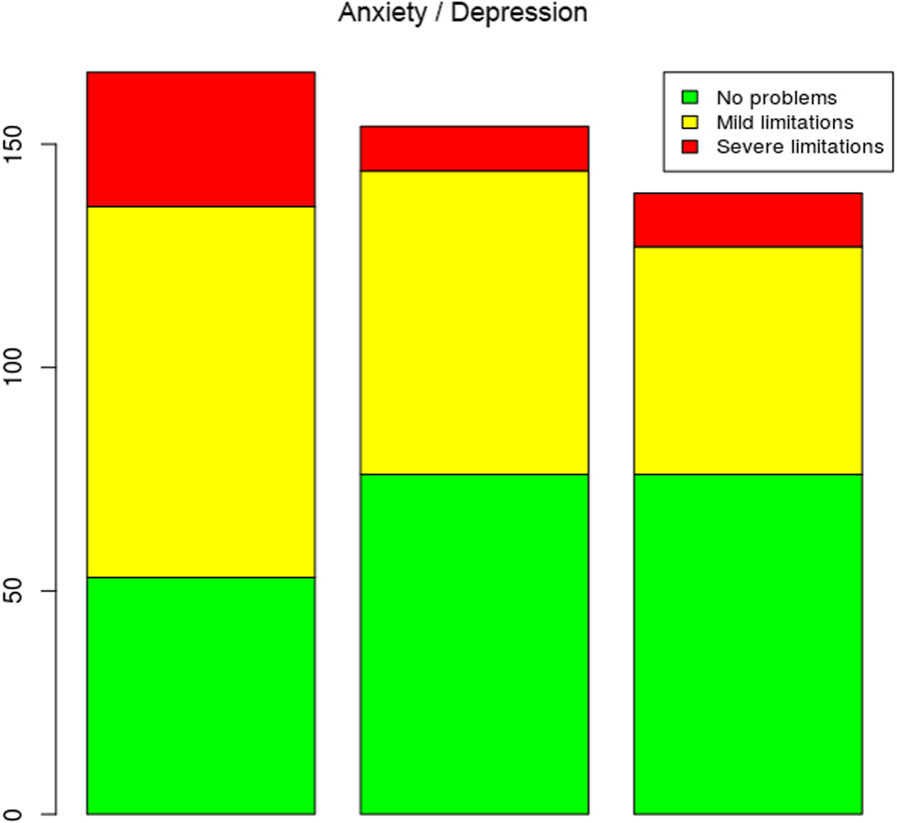
Problems reported in the anxiety/depression dimension at the three assessment times

The assessment of the anxiety/depression dimension showed that, as expected, given the increase in pain and usual activities between times 2 and 3, the levels of anxiety/depression slightly increased (Fig. 6).

The analysis of the results from the assessment of quality of life, according to the EQ-5D-3L assessment, specifically at the three times, shows that such patterns of increase are maintained regarding the type of fracture.

As shown above, the type of fracture persistently affects the assessment of quality of life, although in general, the gains in the five dimensions were considerable.

Given the non-parametric distribution of the outcome variable (quality of life expressed as utility), we compared the distributions using the Friedman Test, with head-to-head comparisons (t_1_ vs. t_2_, t_2_ vs. t_3_), and the Wilcoxon signed-rank test with Bonferroni correction. All comparisons performed showed significant differences (*p* ≤ 0.0005), enabling us to reject the null hypothesis that no differences in variability occurred in the analysis at the three assessment times.

### Calculation of utility measures in the assessment of the quality of life of patients with femoral fractures

Regarding the calculation of utility measures in assessing the quality of life of patients with femoral fractures, 58 different health states were found in the study sample. The results also showed that at the first assessment time, before the surgical procedure, the reported health states were closer to 0, indicating the worst possible health state. Furthermore, the health dimensions with the lowest assessment scores were mobility, self-care and usual activities. However, in the third health state assessment, which corresponded to the time of the second follow-up visit after the surgical procedure, the assigned scores were approximately 0.5 or higher, as outlined in Table 3, where the dimensions with the lowest assessment scores were pain/discomfort and anxiety/depression.

**Table 3:** Measures of utilities and frequency according to assessment time

AV1	Frequency	AV2	Frequency	AV3	Frequency
0.028	49	0.436	13	0.548	10
0.022	27	0.522	11	0.522	9
0.073	15	0.371	9	0.458	7
0.131	14	0.330	8	0.436	7
0.176	12	0.037	7	0.330	6
0.037	11	0.028	7	0.634	6
0.433	7	0.321	6	0.408	5
0.059	5	0.472	5	0.433	5
0.266	4	0.520	5	0.321	5
0.087	4	0.570	4	0.520	4

AV1; AV2 and AV3 means of t_1_, t_2_ and t_3_

In addition, regarding the distribution of utility measures obtained at each assessment, at the first assessment time, upon admission, the utility measures generated were more concentrated (more patients at lower utility levels). Conversely, at the third time, the results were more distributed.

Although measuring the mortality rate was not an objective of this study, a 5.84% 60-day mortality rate was identified, and the mortality rate remained at 6% at the 1-year follow-up period.

## Discussion

One of the most important demographic changes that Brazil experienced at the end of the twentieth century was the marked aging of the population, which resulted in increases in the relative and absolute presence of the population aged 60 and older [17]. This is a key population segment, with life experiences, qualifications and potential to offer to society. Conversely, there are weaknesses and demands specific to this age group.

In this research study, the results showed that, considering the demographic indicators of the sample, most participants were elderly people older than 60 years (60.76%) and women (53.3%). However, considering only the elderly population older than 60 years, the ratio of women increased to 72%, although the ratio increased even more when analyzing the sample population older than 80 years: of the 38 elderly people in this age group, only two were men. These data corroborate the findings of other studies related to femoral fractures [3, 8, 17–26].

In a study conducted by Alffram [25], 30 patients with femoral neck fractures were analyzed, with ages that ranged from 70 to 95 years, with a mean of 83 years, including 24 women (80%) and six men (20%). Chikude, Fujiki, Honda, Ono & Milani [24] also noted the close relationship between femoral fractures and elderly people when conducting a research study in which they determined that the mean age range of patients with femoral neck fractures was 75–80 years among women and slightly younger among men (70–75 years). The authors also indicated that 80% of patients were women, that is, femoral neck fractures were predominantly found among women, in line with the results from the studies conducted by Alffram and from this research study.

Another fact noted in the sample group of the present research study was the high prevalence rate of proximal fractures when dividing patients based on the type of femoral fracture. Confirming the findings of our research, Mesquita and colleagues stated that the increase in the number of elderly people is a fact in our country, highlighting the discussion on debilitating events in this age group, particularly the occurrence of falls, for their physical, psychological and social consequences. A key consequence is the proximal femoral fracture, responsible for a considerable number of surgeries and hospitalizations and representing high costs for the public health system [3].

Accordingly, femoral neck fractures in the elderly play a key role due to both their frequency and their severity because they lead to increases in dependency and mortality by approximately 50% in 1 year [8].

Fractures in the elderly may lead to various types of complications, particularly postsurgical complications, with their most serious consequence being death. Lebrão, Laurenti, de Saúde, Lebrão & Duarte [17] found that 28.6% of elderly people reported “falls”, and their frequency increased with age: 26.2% in those aged from 60 to 74 years and 36.9% in those older than 75 years. Interestingly, in this study, falls were more frequent among women (33%) than among men (22.3%).

Based on the studies conducted by Parker & Hondoll [27], the incidence of proximal femoral fractures significantly increased in recent decades and is expected to double in the next 25 years due to the increase in life expectancy of the population [27]. Thus, we consider that the demographic transition is related to the increase in chronic-degenerative diseases, including osteoporosis.

Regarding previously existing conditions among elderly patients, reported at the time of fracture, the research study identified the following diseases with the strongest effects: hypertension (58%), diabetes mellitus (22%) and heart disease (19%); conversely, heart, lung and kidney disease, stroke and diabetes mellitus were identified in the literature as the comorbidities with the strongest effects on patients with femoral fracture [28]. The percentage of sample participants of that research study with no comorbidity was low, failing to reach 25%.

Thus, according to the selected literature on proximal femoral fractures, the results show that the death rates have been increased by the existence of comorbidities. Their effect on mortality has been measured by both the number of coexisting diseases and their types. Patients with a higher number of coexisting diseases are more likely to die [28].

Transtrochanteric fractures affect the elderly more than intracapsular femoral neck fractures, according to Zuckerman, Skovron, Koval, Aharonoff & Frankel [29] *apud* Guimarães, Guimarães & Franco [30]. The incidence rates of osteoporosis and clinical comorbidities are also higher among these patients. In general, “the fracture pattern is multi-fragmented and unstable, determining, in the first 3 months after the surgical treatment, a mortality rate twice as high as in the group of femoral neck fractures” [28].

In regards to the outcome death, elderly patients with proximal femoral fractures have high mortality rates due to femoral neck fractures. Research studies indicate that the mortality rate ranges from 20 to 30% in the first year after surgical intervention [7, 21, 31–39]. In the papers surveyed, the rates ranged from 7.4 to 34.8%, with a mean mortality rate of 21.8%. These articles found that old age, previous comorbidities and male sex are the most important contributing factors to the occurrence of death among the elderly. However, in the present research study, the percentage of the sample that died was 6%, which was lower than that reported by the aforementioned studies.

Another finding from this research study that differs from the studies surveyed was that 89% patients who died were women, whereas the studies identified in the literature [21, 22, 32] claimed that they found a higher number of deaths among male patients.

With respect to the age group, the results showed a 77% death rate among patients older than 50 years. However, according to Gulhan [40], after 50 years of age, the frequency of this type of fracture in women is twice as high as in males, with percentages of deaths ranging from 15 to 20% within 1 year from the fracture and approximately 50% of survivors needing special, long-term care [33].

Considering the literature examined, the results showed that, although many studies relating the results from the femoral neck fracture treatment to the surgical technique used have been published [40–45], little has been described regarding the postoperative quality of life of patients subjected to surgical treatment.

According to Idler & Benyamini [46], self-reported health assessments are no longer considered merely impressions related to actual health conditions. Several studies have recently shown that individuals reporting poor health conditions have consistently higher mortality risks than those reporting better health states.

Regarding the assessment of quality of life using the EQ-5D-3L at the three time points selected in this research study (admission and first and second follow-up visits), we observed significant gains in quality of life from the first to the third assessment times, with only 2% of the sample with health states translating utility measures above 0.5 at the first assessment time, increasing to 26% at the third assessment time.

However, the results also showed that the gains in quality of life from the second to the third time points in the assessment of the dimensions pain/discomfort and anxiety/depression were smaller than in the other dimensions assessed. Evidence suggests that such changes occurred precisely because of the increase in mobility, partial recovery of self-care capacity and recovery of usual activities. Thus, this recovery half-time may be presumably more painful and generate greater anxiety in patients.

All comparisons of the distributions of results using the Friedman Test, with head-to-head comparisons (t_1_ vs. t_2_, t_2_ vs. t_3_), and the Wilcoxon signed-rank test with Bonferroni correction were significant (*p* ≤ 0.0005), enabling us to reject the null hypothesis that no differences in the variance of the study variable occurred between the three assessment times.

All assessments of the five dimensions of the EQ-5D-3L at the three time points showed gains in quality of life in the significance test based on a liner regression model. However, when performing the test based on several models, the model with the best fit only included the variables surgical infection (SI) and postoperative complications (PC), which were negatively associated with the variation in quality of life. Thus, the model fit explains 9% of the variance of the outcome variable.

Furthermore, the assessment of utility measures based on the health states reported by the study sample showed an upward curve of gains as a function of time in all study dimensions. A total of 35 health states were identified at assessment time 1, 58 health states at assessment time 2 and 56 health states at assessment time 3, with evidence showing a more negative assessment close to 0 at the first assessment time, whereas this measurement resulted in utility measures closer to 1 at the last assessment time.

It should also be noted that the identification of the utility values found based on the composition of health states and the comparison with other studies with longer study periods urged the authors to conduct a new assessment using the EQ-5D-3L and the VAS 1 year after the first assessment of sample patients. That assessment has already been conducted in 40 sample patients by phone call, and only one death was recorded in addition to those that had already been recorded. This reassessment has shown a significant improvement in the outcomes of the sample health states after a longer period, with mortality rates much lower than those found in the research literature.

We acknowledge the main limitation of this study is the data came from a single reference orthopedic hospital which could lead to selection bias. However, we assume that the patients admitted, and quality of care provided in this public hospital do not significantly differ from other public ones in Brazil. Furthermore, the shorter follow-up period we have chosen minimizes losses to follow-up but makes comparability with other studies more difficult. Another limitation is that we did not control for comorbidities. We believe this issue is minimized due to the fact the majority of the sample has at least one comorbidity and we did not wish to over-stratify our analysis due to the fact we wished to present utility values to a broader group of hip fracture patients aiming economic evaluations. As this is an observational study, we cannot conclude that the changes in quality of life over time were caused by the hip fracture evolution itself.

## Conclusions

The use of the EQ-5D-3L as a generic, multi-attribute instrument developed by the EuroQol group to assess gains in quality of life after femoral fracture surgery in a national reference Orthopedic Hospital proved quite useful in recognizing the factors that might affect the quality of life of patients with femoral fractures. Although the population sample of this research study mostly consisted of people older than 60 years, this instrument also proved useful when comparing the recovery of quality of life between young and elderly patients and between men and women with femoral fractures.

Accordingly, the choice of the EQ-5D-3L instrument in the present research study aimed not only to assess the quality of life per se after orthopedic surgery (femoral fracture), providing clinical information, but also to apply the instrument towards supporting future economic evaluation studies of new technologies for femoral fractures.

## Abbreviations

AO/ASIF: Association for Osteosynthesis/Association for the Study of Internal Fixation (OTA)
EQ-5D-3L: EuroQol Group 3-level descriptive system of health-related quality of life states
HRQol: Health-Related Quality of Life
INC: Brazilian National Institute of Cardiology (Instituto Nacional de Cardiologia)
INTO: Brazilian National Institute of Traumatology and Ortopedics (Instituto Nacional de Traumatologia e Ortopedia)
ISOC: International Society of Orthopaedic Centers
NATS: Brazilian Institute for Health Technology Assessment (Núcleo de Avaliação de Tecnologias em Saúde)
OTA: Orthopaedic Trauma Association
QALY: Quality adjusted life year
VAS: Visual analogue scale

## References

[1] Brasil. DATASUS. Informações de Saúde. TabNet. http://www2.datasus.gov.br/DATASUS/index.php. Accessed 3 Feb 2017.

[2] Van Balen R, Steyerberg EW, Polder JJ, Ribbers TL, Habbema JD, Cools HJ. Hip fracture in elderly patients: outcomes for function, quality of life, and type of residence. Clin Orthop Relat Res. 2001:232–43. 11550871

[3] Mesquita GV, Lima M, Santos AMR, Alves ELM, Brito JNPO, Martins MCC (2009). Morbimortalidade em idosos por fratura proximal do fêmur. Texto Contexto Enfermagem.

[4] Lustosa LP, Bastos EO (2009). Fraturas proximais do fêmur em idosos: qual o melhor tratamento?. Acta Ortop Bras..

[5] Pereira SR, Puts MT, Portela MC, Sayeg MA (2010). The impact of prefracture and hip fracture characteristics on mortality in older persons in Brazil. Clin Orthop Relat Res.

[6] Johnston AT, Barnsdale L, Smith R, Duncan K, Hutchison JD (2010). Change in long-term mortality associated with fractures of the hip: evidence from the scottish hip fracture audit. J Bone Joint Surg Br..

[7] Garcia R, Leme MD, Garcez-Leme LE (2006). Evolution of Brazilian elderly with hip fracture secondary to a fall. Clinics.

[8] Barbosa MLJ, Nascimento EFA (2001). Incidência de internações de idosos por motivo de queda em hospital geral em Taubaté. Rev Biocienc.

[9] Fleck MPA, Leal OF, Louzada S, Xavier M, Chachamovich E, Vieira G (1999). Desenvolvimento da versão em português do instrumento de avaliação de qualidade de vida da OMS (WHOQOL-100). Rev Bras Psiquiatr.

[10] AO/OTA. Fracture and dislocation classification. https://classification.aoeducation.org/. Accessed 24 Feb 2016.

[11] Euroqol Group (2000). EQ-5D a measure of health-related quality of life developed by the EuroQol group: user guide.

[12] Parsons N, Griffin X, Achten J, Costa M (2014). Outcome assessment after hip fracture: is EQ-5D the answer?. Bone Joint Res.

[13] Gierstsen J, Baste V, Fevang J, Furnes O, Engesæter L (2016). Quality of life following hip fractures: results from the Norwegian hip fracture register. BMC Musculoskelet Disord.

[14] Brooks R, Rabin R, de Charro F (2003). The measurement and valuation of health status using EQ-5D: a European perspective.

[15] Santos M, Cintra M, Monteiro A (2016). Brazilian valuation of EQ-5D-3L health states: results from a saturation study. Med Decis Mak.

[16] StataCorp Stata 14.2. Stata statistical software. USA. https://www.stata.com/. Accessed 19 Dec 2016.

[17] Lebrão ML, Laurenti R, de Saúde IN, Lebrão ML, Duarte YAO (2003). SABE – saúde, bem-estar e envelhecimento – o projeto sabe no município de São Paulo: uma abordagem inicial.

[18] Rebelatto JR, Morelli JGS (2004). Fisioterapia geriátrica. A prática de assistência ao idoso.

[19] Perracini MR, Ramos LR (2002). Fatores associados a quedas em uma coorte de idosos residentes na comunidade. Rev Saude Publica.

[20] Maciel ACC, Guerra RO (2005). Prevalência e fatores associados ao déficit de equilíbrio em idosos. Rev Bras Ciênc Mov.

[21] Fierens J, Broos PL (2006). Quality of life after hip fracture surgery in the elderly. Acta Chir Belg.

[22] Laranjeiras JA, Ribeiro TA, Guterres LW, Nascimento DZ, Fortes BC, Guerra VA. Estudo prospectivo da mortalidade e morbidade em fraturas do fêmur proximal em pacientes com 65 anos de idade ou mais. In: Anais XV Congresso Sul Brasileiro de Ortopedia Traumatologia - SULBRA; 2007 Jun 21–23; Gramado, Brasil. Gramado: SOTRS. p. 2007.

[23] Hamra A, Ribeiro MB, Miguel OF (2007). Correlação entre fratura por queda em idosos e uso prévio de medicamentos. Acta Ortop Bras..

[24] Suzuki I, Herbert S, Xavier R (2003). Alterações ortopédicas em geriatria. Ortopedia e Traumatologia: princípios e prática.

[25] Alffram PA (1964). An epidemiologic study of cervical and trochanteric fractures of the femur in an urban population. Analysis of 1,664 cases with special reference to etiologic factors. Acta Orthop Scand Suppl.

[26] Chikude T, Fujiki EN, Honda EK, Ono NK, Milani C (2007). Avaliação da qualidade de vida dos pacientes idosos com fratura do colo do fêmur tratados cirurgicamente pela artroplastia parcial do quadril. Acta Ortop Bras.

[27] Parker MJ, Handoll HH. Intramedullary nails for extracapsular hip fractures in adults. Cochrane Database Syst Rev. 2006:CD004961. 10.1002/14651858.CD004961.pub3 16856070

[28] Stolnicki B, Aronson D (1993). Avaliação densitometria em portadores de fraturas osteoporóticas. Rev Bras Ortop.

[29] Zuckerman JD, Skovron ML, Koval KJ, Aharonoff G, Frankel VH (1995). Postoperative complications and mortality associated with operative delay in older patients who have a fracture of the hip. J Bone Joint Surg Am.

[30] Guimarães JAM, Guimarães ACA, Franco JS (2008). Avaliação do emprego da haste femoral curta na fratura trocantérica instável do fêmur. Rev Bras Ortop.

[31] Hebert S, Xavier R, Pardini JAG, Filho TEPB (2003). Ortopedia e traumatologia: princípios e prática.

[32] Souza RC, Pinheiro RS, Coeli CM, Camargo KR, Torres TZG (2007). Aplicação de medidas de ajuste de risco para a mortalidade após fratura proximal de fêmur. Rev Saude Publica.

[33] Röder F, Schwab M, Aleker T, Mörike K, Thon KP, Klotz U (2003). Proximal femur fracture in older patients–rehabilitation and clinical outcome. Age Ageing.

[34] Nurmi I, Narinen A, Lüthje P, Tanninen S (2004). Functional outcome and survival after hip fracture in elderly: a prospective study of 106 consecutive patients. J Orthop Traumatol.

[35] Silveira VAL, Medeiros MMC, Coelho-Filho JM, Mota RS, Noleto JCS, Costa FS (2005). Incidência de fratura do quadril em área urbana do Nordeste Brasileiro. Cad Saude Publica.

[36] Haentjens P, Autier P, Barette M, Venken K, Vanderschueren D, Boonen S (2007). Survival and functional outcome according to hip fracture type: a one-year prospective cohort study in elderly women with an intertrochanteric or femoral neck fracture. Bone.

[37] Pinheiro RS, Camargo KR, Vidal EIO, Coeli CM, Vieira RA (2006). Utilização do SIH-SUS e do SIM para o cálculo da mortalidade hospitalar e em 30 dias para as internações de pacientes com fratura proximal do fêmur. Cad Saude Colet.

[38] Gulhan EG, Pickles B, Compton A, Cott C, Simpson J, Vandervoort A (2000). Osteoporose e fraturas osteoporóticas. Fisioterapia na Terceira idade.

[39] Dzupa V, Bartonicek J, Skála-Rosenbaum J, Prikazsky V (2007). Mortality in patients with proximal femoral fractures during the first year after the injury. Bone.

[40] Calder SJ, Anderson GH, Jagger C, Harper WM, Gregg PJ (1996). Unipolar or bipolar prosthesis for displaced intracapsular hip fracture in octogenarians: a randomised prospective study. J Bone Joint Surg Br..

[41] Davison JN, Calder SJ, Anderson GH, Ward G, Jagger C, Harper WM (2001). Treatment for displaced intracapsular fracture of the proximal femur. A prospective, randomised trial in patients aged 65 to 79 years. J Bone Joint Surg Br.

[42] Faraj AA, Branfoot T (1999). Cemented versus uncemented Thompson’s prostheses: a functional outcome study. Injury.

[43] Follacci F, Charnley J (1969). A comparison of the results of femoral head prosthesis with and without cement. Clin Orthop Relat Res.

[44] Judet J, Judet R (1950). The use of an artificial femoral head for arthroplasty of the hip joint. J Bone Joint Surg Br..

[45] Moore AT, Bohlman HR (1943). Metal hip joint. A case report. JBJS.

[46] Idler EL, Benyamini Y (1997). Self-rated health and mortality: a review of twenty-seven community studies. J Health Soc Behav.

